# The Impact of Curtin University's Activity, Food and Attitudes Program on Physical Activity, Sedentary Time and Fruit, Vegetable and Junk Food Consumption among Overweight and Obese Adolescents: A Waitlist Controlled Trial

**DOI:** 10.1371/journal.pone.0111954

**Published:** 2014-11-06

**Authors:** Leon M. Straker, Erin K. Howie, Kyla L. Smith, Ashley A. Fenner, Deborah A. Kerr, Tim S. Olds, Rebecca A. Abbott, Anne J. Smith

**Affiliations:** 1 School of Physiotherapy and Exercise Science, Curtin University, GPO Box U1987, Perth, Western Australia, 6845, Australia; 2 School of Physiotherapy and Exercise Science, Curtin University, Perth, Australia; 3 School of Psychology and Speech Pathology, Curtin University, Perth, Australia; 4 School of Public Health, Curtin University, Perth, Australia; 5 Health and Use of Time (HUT) Group, University of South Australia, Adelaide, Australia; University of Bath, United Kingdom

## Abstract

**Background:**

To determine the effects of participation in Curtin University's Activity, Food and Attitudes Program (CAFAP), a community-based, family-centered behavioural intervention, on the physical activity, sedentary time, and healthy eating behaviours of overweight and obese adolescents.

**Methods:**

In this waitlist controlled clinical trial in Western Australia, adolescents (n = 69, 71% female, mean age 14.1 (SD 1.6) years) and parents completed an 8-week intervention followed by 12 months of telephone and text message support. Assessments were completed at baseline, before beginning the intervention, immediately following the intervention, and at 3-, 6-, and 12- months follow-up. The primary outcomes were physical activity and sedentary time assessed by accelerometers and servings of fruit, vegetables and junk food assessed by 3-day food records.

**Results:**

During the intensive 8-week intervention sedentary time decreased by −5.1 min/day/month (95% CI: −11.0, 0.8) which was significantly greater than the rate of change during the waitlist period (p = .014). Moderate physical activity increased by 1.8 min/day/month (95% CI: −0.04, 3.6) during the intervention period, which was significantly greater than the rate of change during the waitlist period (p = .041). Fruit consumption increased during the intervention period (monthly incidence rate ratio (IRR) 1.3, 95% CI: 1.10, 1.56) and junk food consumption decreased (monthly IRR 0.8, 95% CI: 0.74, 0.94) and these changes were different to those seen during the waitlist period (p = .004 and p = .020 respectively).

**Conclusions:**

Participating in CAFAP appeared to have a positive influence on the physical activity, sedentary and healthy eating behaviours of overweight and obese adolescents and many of these changes were maintained for one year following the intensive intervention.

**Trial Registration:**

Australia and New Zealand Clinical Trials Registry ACTRN12611001187932

## Background

Adolescents who are overweight or obese are at greater risk of physical and mental health problems both during adolescence and subsequent adulthood [1],[2]. Physical activity, sedentary behaviour and healthy eating behaviours not only contribute to obesity but also have important independent health implications [3]. The primary outcome focus of most interventions for overweight and obese adolescents has been adiposity rather than activity and healthy eating behaviours [4]. Aside from the importance of these behaviours to multiple health issues, including adiposity [3], a focus on weight-related outcomes may have unintended negative psychological consequences [5]. Evidence also suggests that interventions targeting both activity and healthy eating behaviours should be multi-disciplinary and involve families in community settings for sustained change [4],[6],[7]. Whilst the few studies focussed on behavioural outcomes for overweight and obese adolescents have reported some encouraging findings [8],[9],[10], they have either lacked assessments of sustained change beyond immediately post-intervention [11],[12],[13],[14] or lacked objective measures of activity [8],[9],[15]. Additionally, few studies have used detailed dietary assessment methods, such as food records, to describe changes in healthy eating outcomes for adolescents [16].

Curtin University's Activity, Food and Attitudes Program (CAFAP) was a community-based, family-centered behavioural intervention implemented by a multi-disciplinary team of health practitioners. The current study determined the effects of participation in CAFAP on activity and healthy eating behaviours of overweight and obese adolescents and how behaviours were maintained for one year following the intervention. Specific hypotheses tested were:

- Time in sedentary, light, moderate and vigorous physical activity and intake of fruit, vegetables and ‘junk food’ would change following participation in the CAFAP program and changes would be maintained for up to 12 months
- The rate of change of time in sedentary, light, moderate and vigorous physical activity and intake of fruit, vegetables and ‘junk food’ over the wait-list control period would differ from the rate of change over the intervention and maintenance periods.

## Methods

### Study Design

This study was a staggered entry, within-subject, waitlist controlled clinical trial conducted in Western Australia. This design was selected to minimize ethical concerns with withholding treatment in a no treatment control for 18 months [17] and the likely unacceptably high drop-out over multiple assessments over an extended study duration for a no-treatment or minimal standard care control group. Additionally, the within-subject design reduces error variance from individual differences, thus increasing the power with the expected high dropout rates and reduced sample sizes found in previous studies [4],[10],[13],[18]. The trial was registered (Australia and New Zealand Clinical Trials Registry # ACTRN12611001187932) and the protocol published [19]. The protocol for this trial and supporting CONSORT checklist are available as supporting information; see Checklist S1 and Protocol S1. Participants were assessed at six time-points including baseline, 3-months after baseline prior to beginning the intervention (end of waitlist period), immediately following the 8-week intervention (end of intervention period), and at 3-, 6-, and 12-months following the intervention (maintenance periods). Entry into the program was staggered into three waves, beginning in February, May and August 2012 to control for bias from external events and seasonal changes. Follow-up assessments were completed by December 2013. The program was conducted at three sites (two urban and one rural) with a high proportion of low socio-economic status residents. Curtin University Human Research Ethics Committee approved all procedures (HR105/2011). Written informed assent was obtained from adolescents and consent was obtained from parents prior to commencing the study.

### Recruitment And Participants

**Figure 1:**
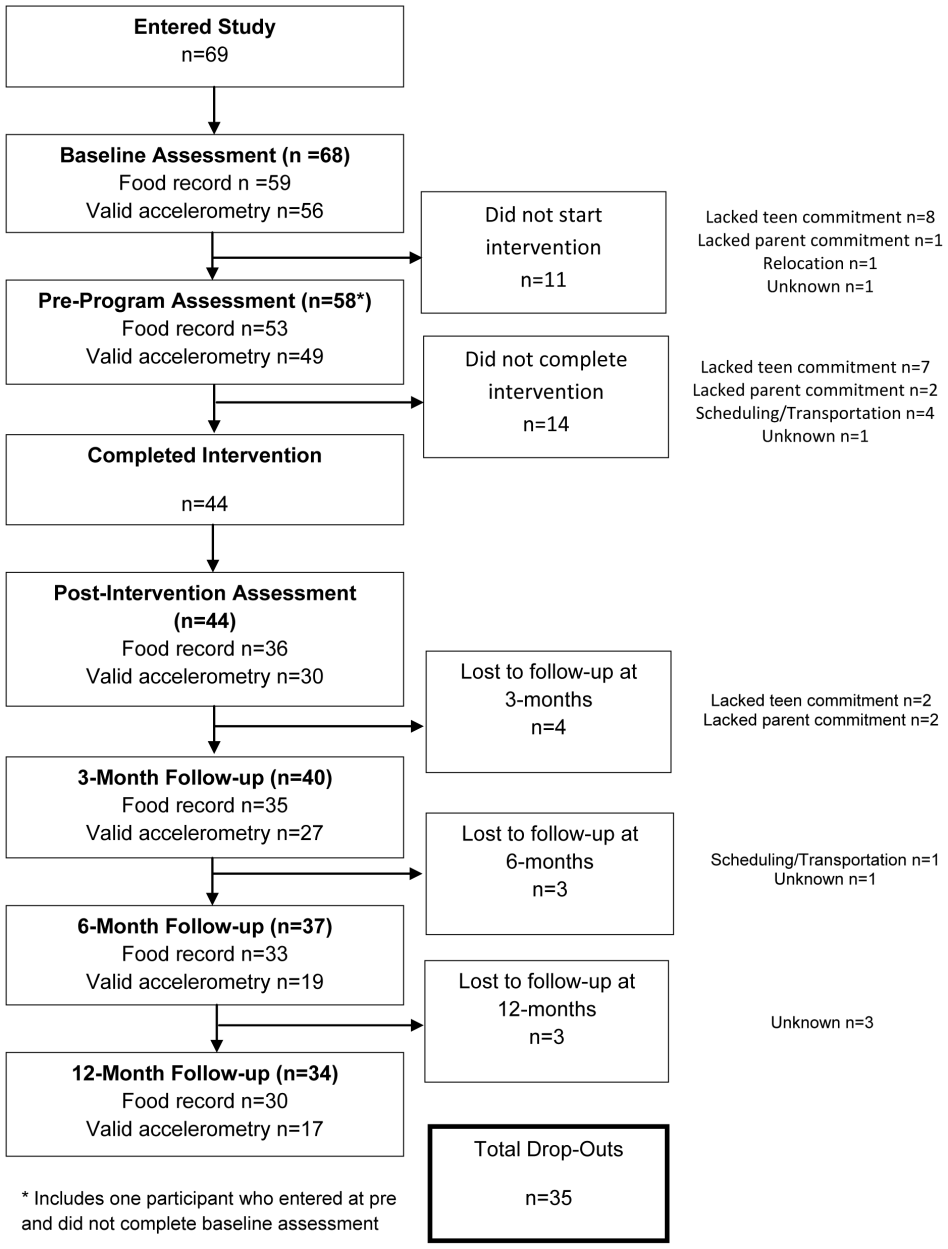
Participant flow diagram for the waitlist controlled trial of Curtin University's Activity, Food, and Attitudes Program.

One hundred and twenty three participants enquired about the program from the community following information provided by health professionals, community newspapers and radio media, and distribution of flyers, of which 69 entered the study. Recruitment began in late November 2011 and continued through to August 2012. To be included in the study, participants had BMI-for-age and sex greater than the 85^th^ percentile on the Centers for Disease Control BMI-for-age growth charts [20] and passed a medical screening prior to participation. Participants were excluded if their obesity was related to a diagnosed metabolic, genetic, or endocrine disease, were receiving current treatment for a psychological disorder, or were unable to attend sessions twice weekly at the community locations. Sample size estimates were reported in the protocol paper [21]. Seven cohorts ranging in size from 6 to 13 adolescents and the same number of parents were conducted in three waves. Recruitment ceased after the planned three waves despite smaller than anticipated numbers due to grant funding constraints. Figure 1 shows the flow of participants through the study.

### Intervention

This intervention was adapted to be conducted in the community from a previous program conducted in healthcare and university settings. Additional detailed formative work with adolescents, researchers and practitioners guided the development and refinement of the curriculum [22]. In summary, a team of 13 multidisciplinary community practitioners (psychologist, physiotherapist/exercise physiologist, dietitian) were trained to implement the program across the seven cohorts, with one facilitator from each domain working with each cohort typically. The theoretical framework for the intervention was self-determination theory [23] and goal setting theory [24] and is described in detail elsewhere [25]. Both instructors and parents were encouraged to provide a need-supportive environment to increase adolescents' autonomous motivation for physical activity and healthy eating behaviours.

The initial 8-week intervention included 2-hour group sessions twice per week in community locations. Both adolescents and parents participated in all sessions, with each session having separate and joint activities. An outline of the sessions and which facilitators supported each session is provided in Table 1. Phone contact was made with participants who missed a session to increase attendance adherence. At every session, adolescents participated in 45-minutes of enjoyable physical activity and a behavioural component on healthy eating, activity and overcoming barriers (see Table 1). During the CAFAP activity sessions, participants were taught to self-monitor their heart rate and self-perceived level of exertion. The sessions began with a warm up, usually 1–2 group games, to increase the heart rate and body temperature in preparation for the circuit exercises. During the circuit training, participants moved through alternate ‘huff and puff’ and ‘strength’ stations. ‘Huff and puff’ stations, like boxing, were designed to increase the participants' heart rate and increase their level of cardiovascular exertion. ‘Strength’ stations included a focus on different parts of the body, specifically trunk (e.g. oblique crunches), upper limb (e.g. bicep curls) and lower limb (e.g. squats). Sessions usually ended with another group game and cool-down stretches. Adolescents were encouraged to bring their own music to play for the group during the activity sessions. Adolescents set weekly, manageable goals and parents were guided to set their own goals to support these adolescent goals.

**Table 1:** Overview of CAFAP sessions.

Session#	Adolescents	Parents
1	^All^Introduction to program – overview of program, introductions, group rules, key messages: be more active, be less inactive, eat more fruit and vegetables, eat less junk food, set goals	^All^Introduction to program – joint session
	^PE^Introduction to Activity sessions – types of activity, benefits, how to assess moderate intensity, warm up, circuit, cool down	^D^Expectations – Parent discussion of their expectations of the program
2	^PE^Activity session – adding stations, review heart rate	^D^Walk and talk (parents and facilitators go for a walk together to model being more active) –topics – get to know each other
	^D^Healthy eating – energy balance, basic nutrition principles: variety and nutrients.	^D^Healthy eating - joint session
3	^PE^Activity session – adding new circuit stations	^P^Understanding adolescence –teens need choice, competence, belonging Parents can provide structure, be involved, support teenager choices
	^PE^Healthy activity – be more active, be less inactive, benefits of being active, activity and inactivity, energy out balance	^PE^Health activity - joint session
4	^PE^Activity session – adding stations	^P^Providing structure – Setting up house rules, monitoring behaviour and observance of rules, consequences for breaking rules.
	^D^Portion size –portion size guidelines, food group intake, eat more fruit and vegetables, eat less junk food	^D^Portion size - joint session
5	^PE^Activity session – increasing speed	Parent introduction to goal setting – setting goals to support teenager goals.
	^P^Teens introduction to goal setting – how to set goals, feedback on current activity and eating behaviours (using pre-intervention assessment information), start to set goals	Walk and talk – topics – get to know each other, how it's going, review house rules
6	^PE^Activity session – increasing speed (30 mins only)	^D^Fast food and dinner- fast food (takeaway) and parent planning for ‘fast’ dinners at home (4:30–5:30pm)
	^All^Teens setting goals (1.5 hours)	^All^Parents setting goals to support teens (last ½ hour only- with teens)
7	^PE^Activity session (30 mins only)	^P^Review and debrief of progress – supporting teenager activity and food goals and competence
	^PE^Goals- (30 mins) - teens reflect on goal progress, write new weekly goals (20 mins) and share with parents (10 mins)	^P^Goals - (20 mins) - 10 mins to review weekly progress then join with teens for 10 mins
	^PE^Family activity– review of key activity messages, benefits of activity, identification of positive and less positive activity, inactivity & sleep habits of family, opportunities for goals	^PE^Family activity – joint session
8	^PE^Activity session	^D^Walk and talk – topics – parenting issues
	^D^Family food – review of key food messages, identification of positive and less positive food habits of family members, encourage positive eating behaviours to use in goal setting	^D^Family Food – joint session
9	^PE^Activity session – (30 mins)	^P^Parent-teen relationships – parenting styles to provide structure, be involved, support teenager choices, maintaining good relationships.
	^PE^Goals – (30 mins) - teens reflect on goal progress, write new weekly goals (20 mins) and share with parents (10 mins)	^P^Goals - (15 mins) - 5 mins to review weekly progress then join with teens for 10 mins
	^P^Overcoming barriers to achieve goals – things that help, influence of mood	^P^Overcoming barriers to achieve goals – joint session
10	^PE^Activity session 45 minutes then teens complete checklist and knowledge and skills mastery check	^D^Walk and talk
	^D^Snacks- problem solving, snacks that help you eat more fruit and vegetables and eat less junk food	^D^Snacks – joint session
11	^PE^Activity session- 30 minutes	^D^Food budgeting – money spent on food groups, planning to get more good food for your money
	^PE^Goals – (30 mins)- teens reflect on goal progress, write new weekly goals (20 mins) and share with parents (10 mins)	^D^Goals - (15 mins) - 5 mins to review weekly progress then join with teens for 10 mins
	^D^Food labelling – understanding labels, sugar in drinks, eat less junk	^D^Food labelling – joint session
12	^PE^Activity session	^P^Community opportunity – ideas for healthy activity and good food in your community using family findings from resource homewor
	^P^Togetherness – creative reflection on program involvement using paint colours to symbolise what participants have gained	^P^Togetherness – joint session
13	^PE^Activity session	^D^Supermarket visit – nutrition and cost of foods, reviewing food labelling skills and budgeting skills
	^P^Teen problem solving to achieve goals – (35 mins) - dealing with setbacks	
	^P^Goals – (25 mins) - teens reflect on goal progress, write new weekly goals (15 mins) and share with parents (10 mins)	^D^Goals - (15 mins) - 5 mins to review weekly progress then join with teens for 10 mins
14	^PE^Activity session	^D^Parents in the kitchen - cooking healthy snacks with fruits and vegetables
	^D^Teens in the kitchen - cooking healthy snacks with fruits and vegetables	^P^Parent problem solving to achieve goals - dealing with setbacks
15	^PE^Activity session – (55 mins)	^D^Recipe ideas – modifying recipes to be tasty and healthy, feedback on program to date
	^PE^Goal review – (5 mins) - Reflect back on program goal set at the beginning	^D^Goals- (10 mins) - 5 mins to review weekly progress and prep for 3month goals for 5 mins
	^All^3 month goal setting. Long term goals. (15 mins just teens)	^All^3 month goal setting – (15 mins) - Follow up/support requirements for post program, Joint session (join after 15 mins)
16	^PE^Activity session – confirm follow up arrangements	^D^Cooking Celebration Preparation- prepare celebration food, confirm follow up arrangements
	^All^Cooking Celebration - cooking healthy party foods	^All^Cooking Celebration – joint session

Session facilitated by: All, physiotherapist/exercise physiologist (PE), dietitian (D) or psychologist (P).

Shading indicates adolescent and parent joint sessions.

During the 12-month follow-up, participants received structured telephone and text message contact at a decreasing frequency based on the same theoretical principles and key messages as during the intensive face-to-face contact period. Contact was based on self-determination theory and goal setting theory and focused on eating more fruits and vegetables, eating less junk food, being less sedentary and being more active. The structuring of text messages is described in detail elsewhere [26]. The phone coaching was completed by members of the facilitation/assessment team who were well known to participants and aimed to provide structure, support attempts at change, and promote adolescents' sense of autonomy. A protocol for adverse events was in place, but no adverse events were reported. Program fidelity was assessed on several occasions for each site by independent observation.

Further details about the intervention, including resources for health professionals wishing to conduct similar programs, are provided at the study website: http://cafap.curtin.edu.au/.

### Measures

The primary outcomes were time in sedentary, light, moderate and vigorous activity and serves of fruit, vegetables and ‘junk food’ [21].

#### Activity

Adolescents were instructed to wear Actical monitors (Respironics; Bend, Oregon, USA) on an elastic band on their right hip for all waking hours for 7 days except for during water-related activities. Data were collected in 15-second epochs. Diary information and visual inspection of the data were used to determine individual daily wear-time. Periods where participants attended CAFAP sessions or assessments were excluded from the data. Accelerometry data were processed using a customised LabView V7 (National Instruments, Austin, TX, USA) program to determine daily minutes of sedentary, light, moderate and vigorous physical activity using intensity cut-points for children [27]. Participants were included in the primary analysis at each assessment period if they had at least three days of at least 8 hours of wear-time [28].

#### Food Intake

Dietary intake was assessed using a 3-day food record including one weekend day. Adolescents were trained in completing the record and estimating portion size using household measures. The completed food record was reviewed by the research dietitian and details were clarified with the adolescent. The records were analysed for the number of serves of fruits and vegetables, determined according to the Australian Guide to Healthy Eating serve sizes [29], and junk food according to published criteria [29],[30]. Serves of fruit (150 g fresh fruit and 30 g dried fruit) were calculated excluding fruit juice, given the difficulty in identifying juice that had been sweetened and the propensity to consume excessive amounts of juice. Vegetables (75 g) included all vegetables other than fried potato, which were included in the junk food serves. The term ‘junk food’ was used to describe foods that are considered energy-dense, nutrient-poor foods [29],[30] and do not belong to the core food groups. To account for the typically high prevalence of underreporting in self-reported food intake [31], underreporting ratios were calculated. Total energy expenditure was estimated from resting energy expenditure calculations [32], and activity energy expenditure from individual participant accelerometer data [33]. When accelerometer data was missing, energy activity energy expenditure was calculated for a sedentary individual [33]. A ratio of reported energy intake from diaries and total energy expenditure (rEI:TEE) was calculated and included as a continuous variable in the analyses [34],[35].

#### Other Measures

Participants completed surveys of basic demographic information and anthropometric measurements were taken at each assessment at the community facility where the program was delivered. Weight and height were measured and used to calculate age and sex adjusted BMI z-scores [36]. Staff assessing outcomes were not blind to the participant's intervention stage, but did not have access to participant prior scores. Participants were not able to be blinded to the intervention. Data on adolescent fitness, food behaviours, perceived parent need-supportive behaviours, autonomous motivation, mental health and quality of life; parent mental health, autonomous motivation to support adolescents, demonstration of need-supportive behaviours, and family functioning; adolescent and parent perceptions of facilitator support; program fidelity; and adolescent, parent and facilitator perceptions of the program were collected and are being reported in detail elsewhere.

### Statistical Analyses

Data were visually inspected for potential outliers. Outliers were checked for data entry errors and corrected where applicable, and biologically implausible values were removed. Data were screened for normality using histograms and multiple measures of location.

Descriptive statistics were calculated (means and standard deviations) at each time point. A comparison of baseline values between those participants completing both the intervention and maintenance periods were compared with those not completing either the intervention or maintenance periods using ANOVA and Χ^2^.

To assess within-subject change, changes in physical activity were assessed using separate linear mixed models with random intercepts for sedentary, light, moderate and vigorous physical activity and adjusted for accelerometer wear-time. Participants who participated in at least 2 assessments were included in statistical models (total used in analysis, n = 56). Missing values were accounted for in the linear mixed models, which uses a likelihood-based estimation procedure resulting in non-biased estimates by imputation of missing responses based upon the surrounding responses and modelled covariance structure. Count data of servings of fruit, vegetables, and junk food were analysed using negative binomial regression utilising generalized estimating equations, with an exchangeable correlation structure and robust estimates of standard errors. Due to the bias that is likely to result from excluding underreporters [35], the ratio of estimated energy expenditure and reported energy intake was included in the models for food servings as a time-varying covariate [34]. Linear contrasts are presented as incidence rate ratios (IRR). Model fits were assessed through residual plots and diagnostics.

In all models, a priori linear contrasts compared the mean point estimate at each time point to pre-intervention point estimates. Further, the monthly rates of change over the following periods were estimated: the waitlist period between baseline and pre-intervention, the intervention period between pre-intervention and post-intervention, and the maintenance period between post-intervention and 12-months. Due to the waitlist control design, changes over the intervention and maintenance periods were compared to changes during the waitlist period. The change over periods was adjusted for varying time between assessments by expressing changes as rate of change per month rather than absolute change across the period. No explicit control for multiple comparisons was performed, rather 95% confidence intervals for all parameter estimates are presented together with actual p-values where appropriate. All analyses were conducted using Stata/IC 13.0 for Windows (StataCorp LP, College Station TX, USA).

## Results

### Baseline Characteristics

Adolescents who completed the study were comparable to those who dropped out before completing the intensive intervention or during the maintenance phase except for the average number of days wearing the accelerometer and daily minutes of moderate physical activity (Table 2).

**Table 2:** Baseline characteristics of adolescents in Curtin University's Activity, Food, and Attitudes Program in total sample and those who did and did not complete the program (n, % or Mean (SD)).

	Total	Did not complete intervention	Did not complete maintenance	Completed	P-Value
n	*68* *	*25*	*10*	*34*	
Gender (% female)	71.0	64.0	60.0	79.4	0.31
Age (years)	14.1 (1.6)	14.6 (1.7)	13.4 (1.4)	13.9 (1.5)	0.10
Height (cm)	162.9 (8.6)	164.4 (9.4)	161.4 (7.4)	162.2 (8.5)	0.54
Weight (kg)	87.8 (20.4)	89.0 (22.5)	81.5 (14.5)	88.7 (20.5)	0.59
BMI (kg/m2)	32.8 (6.3)	32.6 (6.5)	31.2 (4.7)	33.5 (6.6)	0.59
BMI z-score	2.1 (0.4)	2.0 (0.5)	2.1 (0.3)	2.2 (0.4)	0.60
n (valid accelerometry)	*56*	*16*	*8*	*32*	
**Mean days worn**	**6.1 (1.3)**	**5.6 (1.3)**	**5.4 (1.2)**	**6.6 (1.1)**	**0.01**
Mean weartime (min/day)	779.0 (74.3)	758.3 (69.5)	759.8 (94.3)	794.2 (70.0)	0.21
Sedentary (min/day)	547.4 (91.7)	537.1 (90.1)	510.1 (111.6)	561.8 (86.8)	0.32
Light PA (min/day)	197.7 (54.0)	181.2 (43.4)	221.2 (61.2)	200.0 (53.5)	0.22
**Moderate PA (min/day)**	**32.6 (18.4)**	**38.7 (21.5)**	**27.8 (11.6)**	**30.7 (17.9)**	**0.02**
Vigorous PA (min/day)	1.5 (2.6)	1.4 (6.3)	0.7 (0.9)	1.7 (3.1)	0.11
N (valid food record record)	*58*	*16*	*10*	*32*	
Fruit (servings/day)	0.8 (0.8)	0.8 (1.0)	0.4 (0.3)	0.8 (0.8)	0.29
Vegetables (servings/day)	1.2 (1.0)	1.3 (1.1)	1.2 (1.2)	1.2 (1.0)	0.96
Junk food (servings/day)	5.8 (3.5)	6.4 (4.6)	5.0 (1.8)	5.7 (3.4)	0.60
Energy (kJ/day)	8101.1 (2503.0)	8405.8 (2807.0)	7236.8 (1854.6)	8215.3 (2530.1)	0.48

*One participant did not complete baseline testing but entered the study during the waitlist period.

P-values for ANOVA comparison between three groups: Did not complete intervention, Did not complete maintenance, Completed.

Variables with significant group differences highlighted in bold.

### Changes In Physical Activity And Sedentary Time

**Figure 2:**
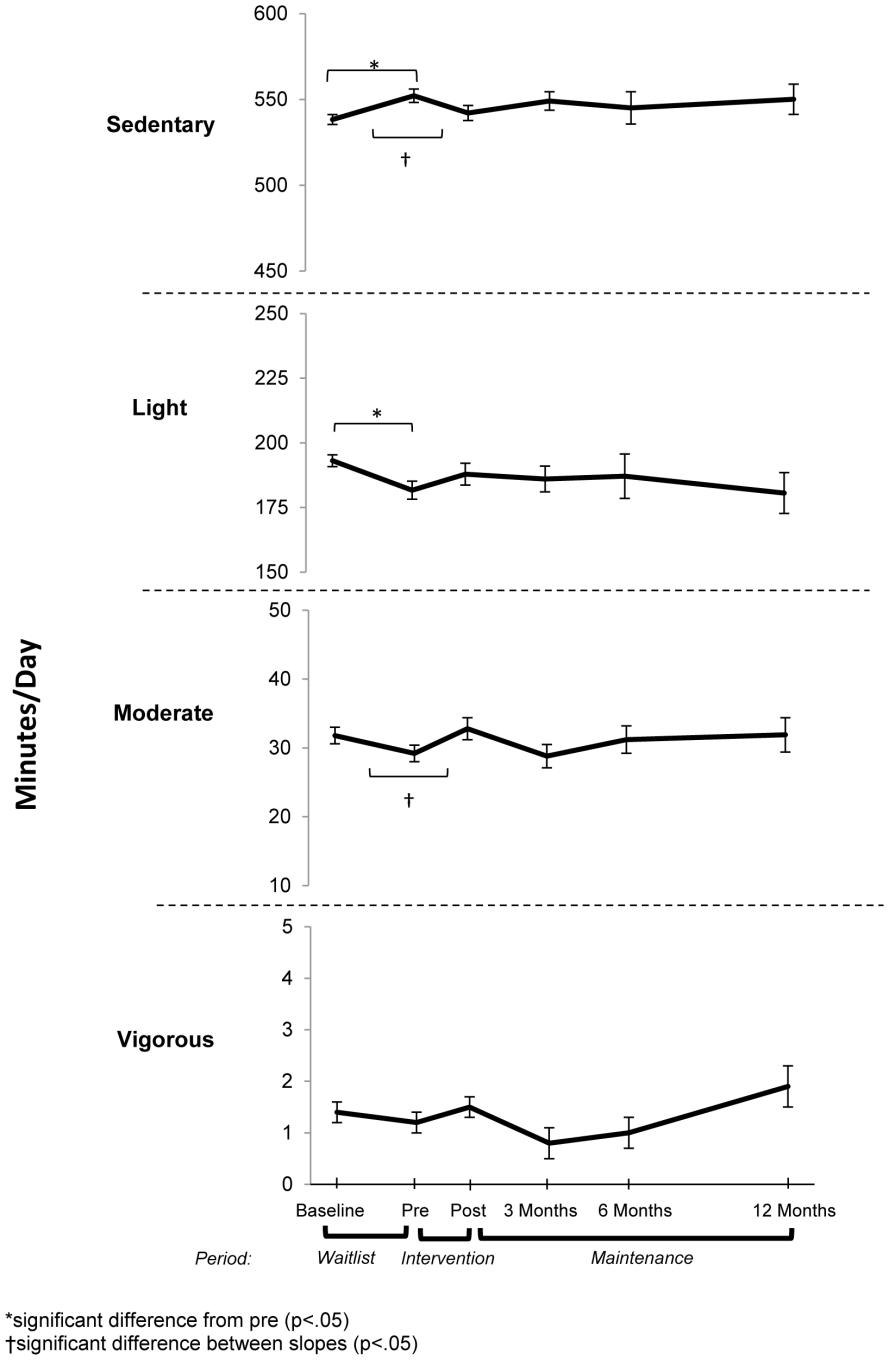
Mean (+ standard error) changes in activity by intensity (results from mixed models).

The changes in physical activity at each time point and the rate of monthly change over each period can be seen in Table 3. From baseline to pre-intervention (the waitlist period), there was a significant increase in the point estimate of sedentary time and a decrease in light physical activity as seen in Figure 2. Moderate and vigorous physical activity did not significantly change during the waitlist period.

**Table 3:** Mean (95%CI) physical activity and healthy eating point estimates and rates of change across the study.

	Point Estimates						Rate of Change		
	Baseline	Pre	Post	3- months	6- months	12- Months	Waitlist Period	Intervention Period	Maintenance Period
							*(Baseline to Pre)*	*(Pre to Post)*	*(Post to 12-months)*
	*Minutes per day (SE)*						*Mean Δ per month, min per day (95% CI)*		
**Sedentary^a^**	**532.3** (3.3)	548.2 (3.7)	537.9 (4.2)	544.8 (5.3)	540.9 (9.1)	545.8 (8.3)	5.3 (1.8, 8.8)	**−5.1** (−11.0, 0.8) †	0.7 (−0.8, 2.2)
**Light**	**199.7** (2.7)*	186.4 (3.5)	192.8 (4.1)	190.9 (4.9)	191.9 (8.7)	185.5 (8.1)	−4.4 (−7.6, −1.2)	3.2 (−2.5, 8.9)	−0.6 (−2.0, 0.8)
**Moderate**	31.1 (1.3)	29.4 (1.1)	33.0 (1.5)	29.0 (1.7)	31.4 (2.0)	32.1 (2.4)	−0.9 (−2.1, 0.3)	**1.8** (−0.04, 3.6) †	−0.1 (−0.5, 0.4)
**Vigorous**	1.5 (0.2)	1.3 (0.2)	1.6 (0.2)	0.8 (0.3)	1.0 (0.3)	2.0 (0.4)	−0.1 (−0.3, 0.1)	0.1 (−0.1, 0.4)	0.04 (−0.04, 0.1)
	*Servings per day (SE)*						*Monthly incidence rate ratio (95% CI)*		
**Fruit^b^**	0.8 (0.1)	0.6 (0.1)	**1.1** (0.2)*	**1.1** (0.1)*	**0.9** (0.1)*	**1.0** (0.2)*	0.94 (0.86, 1.03)	**1.33** (1.11, 1.60) †	0.99 (0.97, 1.02)
**Vegetables**	1.3 (0.2)	1.3 (0.1)	1.3 (0.2)	1.4 (0.2)	**1.7** (0.2)*	1.4 (0.2)	1.00 (0.91, 1.10)	1.00 (0.85, 1.18)	1.01 (0.98, 1.03)
**Junk Food**	4.6 (0.3)	4.6 (0.4)	**3.2** (0.3)*	**3.4** (0.3)*	**3.3** (0.4)*	4.3 (0.5)	1.00(0.95, 1.06)	**0.83** (0.74, 0.94) †	1.02(1.00, 1.05)

a. Activity variables estimated from mixed models with random intercepts adjusted for wear-time

b. Healthy eating variables estimated from negative binomial regression using general estimating equations with random intercepts adjusted for underreporting ratio

*significant difference from Pre point estimate (p<.05)

† significant difference in the rate of change compared to Waitlist Period (p<.05)

Following the intensive 8-week intervention there was a small but non-significant reduction in the point estimate of sedentary time but the rate of change in sedentary time over the intervention period was significantly different to the rate of change over the waitlist period, indicating a decrease in sedentary behaviour during the intervention period. There were small but non-significant differences in light, moderate and vigorous point estimates after the intervention. The rate of change in moderate activity over the intervention period was significantly different to the rate of change during the waitlist period, indicating an increase in moderate activity during the intervention.

During the entire 12-month maintenance period there were only small changes in physical activity and sedentary time.

### Changes In Servings Of Fruit, Vegetables, And Junk Food

Servings of fruits, vegetables and junk food did not change significantly from baseline to pre-intervention as seen in Table 3.

**Figure 3:**
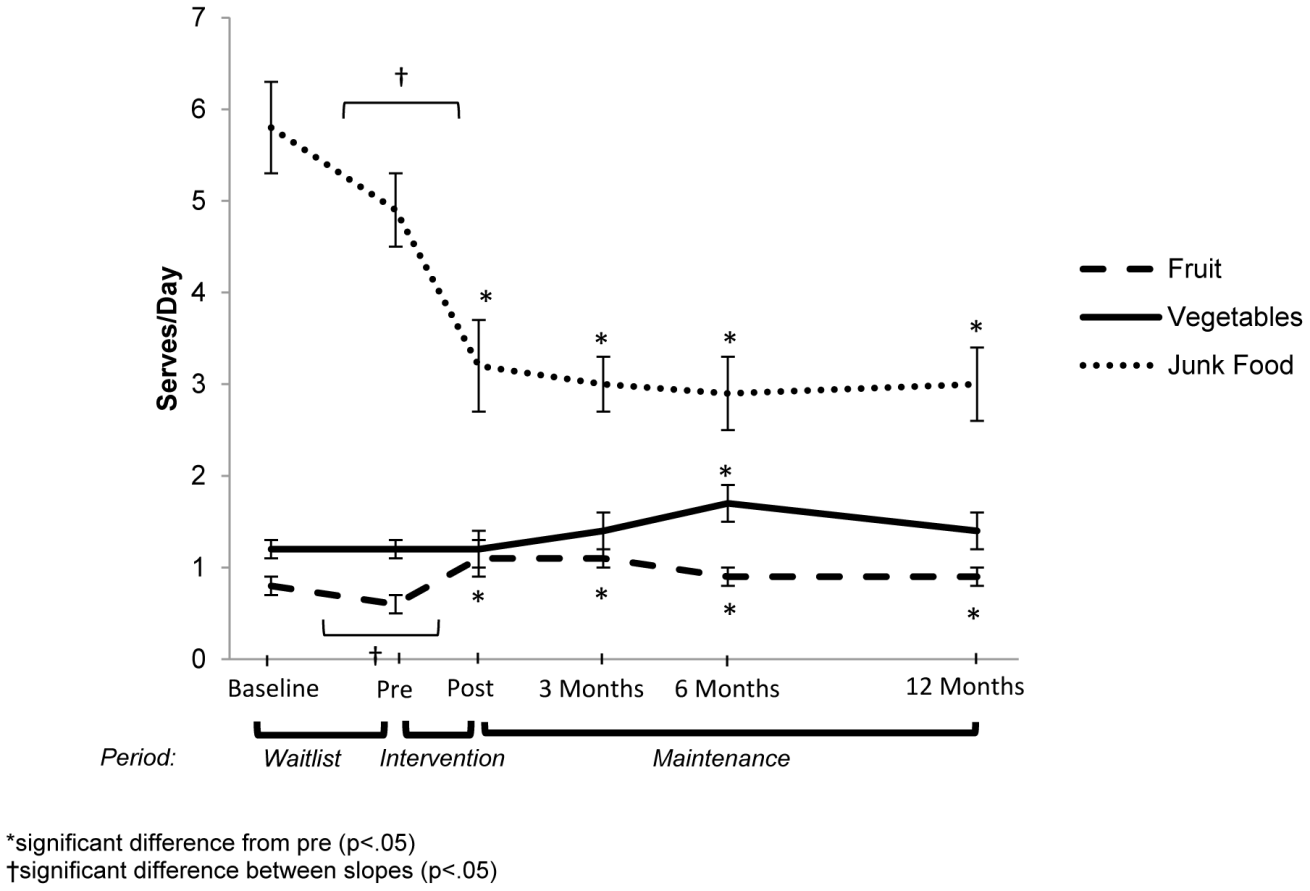
Mean (+ standard error) changes in servings of fruit, vegetables and junk food (results from negative binomial regression).

Following the intensive intervention, there was a significant increase in the point estimate of servings of fruit and the rate of change in fruit consumption during the intervention period was significantly different to the rate of change over the waitlist period, indicating an increase in fruit consumption (see Figure 3). There was no significant change in vegetable consumption during the intensive intervention period. Following the intervention, the point estimate of servings of junk food decreased and the rate of change during the intervention period was significantly different to the rate of change over the waitlist period.

During the entire 12-month maintenance period, there were minimal changes in servings of fruit, vegetables and junk food, except the point estimate of vegetable consumption was higher at 6-months compared to pre-intervention.

### Secondary Outcome: Bmi Z-Scores

BMI z-scores did not change significantly during the waitlist or intervention periods but the point estimates at 3-months (2.05, SE 0.02), 6-months (2.03, SE 0.02), and 12-months (2.03, SE.04) were lower (p = .035, p = .042, p = .060 respectively) than at pre-intervention (2.11, SE 0.02). The rates of change in BMI z-scores were not different between the waitlist period (−0.004 per month, 95% CI: −0.02,.01), intervention period (−0.01 per month, 95% CI: −0.04, 0.01) and maintenance period (−0.005 per month, 95% CI: −0.01, 0.002).

## Discussion

Overweight and obese adolescents participating in CAFAP were able to make small, statistically significant and potentially clinically useful improvements in physical activity, sedentary time and healthy eating behaviour trajectories and maintained many of these changes for one year following the intensive intervention. This was the first objective assessment of a community-based family-centered intervention for overweight and obese adolescents focussed on behaviours as the primary outcomes.

### Physical Activity And Sedentary Time

Physical activity decreased during the 3 month waitlist period whilst sedentary time increased. This trend was in the same direction but of greater magnitude than previous findings on the typical activity trajectory during adolescence [37],[38] and may indicate measurement reactivity or anticipation of the impending intervention. A review of longitudinal studies on physical activity across adolescence found physical activity to decrease an average of 7 percent per year [38]. In a time-use survey of over 6,000 Australian adolescents, moderate-to-vigorous physical activity decreased an average of 13–17 minutes per day per year [37]. In light of these findings, simply maintaining levels of physical activity and sedentary time during adolescence should be considered a positive outcome for interventions [38].

The CAFAP intervention was effective at halting and potentially reversing the negative developmental trajectories of increasing sedentary time and decreasing moderate physical activity. Indeed the effect size of 4 minutes a day more moderate to vigorous activity when comparing the waitlist and intervention periods is consistent with activity interventions across child and adolescent samples [39]. Of the few physical activity interventions for overweight and obese adolescents that have measured physical activity by accelerometry, most have found no significant changes [12],[14],[18]. Two similar lifestyle interventions, used self-report to assess physical activity and found limited changes in specific activities [8],[13],[40],[41]. While the change observed in the current study is comparable to that found in other successful interventions, and of similar magnitude to the 10% change anticipated in the *a priori* power calculations [19], there is insufficient evidence from both adult and child samples regarding the specific health implications of a change in this magnitude. Even following the intervention, on average participants were only achieving half of the recommended 60 minutes of MVPA per day [42], with just six percent of participants achieving an average of 60 minutes per day at post-intervention and the 12-month follow-up. However, as previous researchers have suggested, preventing a further decline in already low activity levels may be a key target during this age transition [38], and participating in 30 minutes of activity per day or 150 minutes per week in adulthood has been shown to have widespread health effects [43].

This was the first study to objectively measure physical activity up to 12 months following a lifestyle intervention for overweight and obese adolescents. The maintenance period was less effective at improving physical activity and sedentary time. While the text messaging during this 12 month period to support this behaviour was based on the limited available evidence, little is known on how adolescents actually change their behaviour and respond to such messages [26].

### Healthy Eating

Compared to research on physical activity levels during adolescence, even less is known on how food behaviours typically change during this life stage [44], partially due to the difficulty in obtaining high quality food record data from adolescents.

During the intervention, servings of junk food decreased and servings of fruit increased. There were no changes in vegetable consumption. Evidence from studies in children suggests that fruit consumption may increase more in response to intervention than vegetable consumption [15]. While other studies have reported increases in selected measures of diet or macronutrients [8],[41], only one previous study has used food recalls to measure changes in fruit, vegetable, or junk food intake in overweight and obese adolescents. One program [10] increased combined fruit and vegetable consumption by 0.6 servings per day after 6 months of intervention, similar to the 0.5 servings per day increase in fruit in CAFAP. Using a 15-item food frequency questionnaire, the Loozit study found an improved proportion of participants meeting fruit and vegetable guidelines after a two-month intervention [41]. While the consumption of fruits and vegetables following participation in CAFAP still fell short of Australian guidelines for 2 serves of fruit and 5 serves of vegetables each day, [29] the increase of half a serve of fruit per day almost doubled the amount of fruit consumed by participants at baseline. The magnitude of change was also much greater than the 10% modelled *a priori* [19] and the changes observed in activity. Evidence on the precise health impact of a change of this magnitude given baseline levels of fruit, vegetables and junk food is lacking and thus an important topic for future research.

In the CAFAP intervention, positive changes in fruit and junk food consumption were successfully maintained up to 12 months following the intervention, however, junk food had increased at 12 months. Additionally, vegetable consumption increased 6 months after the intervention. The Loozit study found no changes in fruit, vegetable, or junk food consumption after 12 or 24 months following the intervention [8],[40]. CAFAP focused on behaviours instead of weight loss, which may have contributed to the maintained changes in healthy eating seen in the current study.

### Limitations

The assessment utilised objective measures of physical activity and detailed 3-day food records. While underreporting is known to be a problem of food records, particularly in overweight and obese adolescents [31], they remain the best available method for detecting short-term changes in diet and intake patterns [45]. Additionally, the energy expenditure estimates from accelerometry were compared to reported energy intake, underreporting was consistent throughout the study and the underreporting ratio was included in the statistical models [34],[35].

The study assessments were taken at multiple time-points during a year-long maintenance period, addressing a noted paucity of studies with follow-up of sustained behaviours beyond immediately post-intervention [4],[15]. As is common with many adolescent interventions [4],[10],[13],[18], there was high attrition through the study but analyses were performed on all participants with two time points of data, and sensitivity analyses confirmed findings in those who completed the study. A process evaluation of the intervention is currently underway to explore barriers to successful completion.

While this quasi-experimental study did not have a concurrent control group, the waitlist period provided a within-subject control period comparison for changes seen in the outcomes across the intervention and maintenance periods. This design was selected as providing the best evidence enabling high external validity (staggered entry) and high internal validity (within person control period) whilst providing best practice health behaviour interventions for high-risk adolescents with a 12 months follow-up. For the physical activity and sedentary behaviour outcomes, the changes during the waitlist period were greater than previously published [37],[38], but were in the anticipated direction. The lack of literature on dietary trends across adolescence does not provide a reference for changes in healthy eating outcomes. Whilst there may have been some reactivity during the waitlist period, changes during the intervention were greater than those seen during the waitlist period, suggesting positive effects of the intervention on moderate physical activity, sedentary time and servings of fruit and junk food.

While behaviours are critical outcomes, further research is needed to determine whether such changes in behaviours translate into changes in fitness, mental and physical health status and quality of life. Further research to understand the patterns for both healthy eating behaviour (such as which meals or specific foods are best to target) and physical activity (such as which day of the week or types of physical activities are best to target) could inform more effective interventions, as could a process evaluation of the current study. Research should also explore whether the effects observed were due to the theoretical mechanism proposed [25], that is, whether training parents in need-supportive behaviours can enhance adolescent autonomous motivation and subsequent physical activity and healthy eating behaviours.

The delivery of the program by community health professionals in community settings, whilst challenging, provided high external validity for the findings to be replicable. However, the small sample size and delivery of the intervention in just three sites of similar populations suggest caution in extrapolation to other samples with different social and other characteristics.

## Conclusions

This study found that a community-based, family focussed multi-disciplinary physical activity and healthy eating intervention can have a positive influence on behaviours in overweight and obese adolescents and many of these changes can be maintained for up to a year following the intervention. This is encouraging, especially when contrasted with the common pessimistic trajectories of physical activity and healthy eating during adolescence. Improving physical activity and healthy eating behaviours during adolescence is important for current and future physical and mental health and thus successful programs should be made widely available to benefit as many adolescents as possible.

## Supporting Information

Checklist S1 **CONSORT Checklist.**
(DOCX)

Protocol S1 **Protocol submitted to Curtin Ethics Committee.**
(DOCX)
