# Supplementary material for: The Impact of Curtin University's Activity, Food and Attitudes Program on Physical Activity, Sedentary Time and Fruit, Vegetable and Junk Food Consumption among Overweight and Obese Adolescents: A Waitlist Controlled Trial
Source: PLoS One. 2014 Nov 6;9(11):e111954. doi: 10.1371/journal.pone.0111954 (PMC4222962; doi:10.1371/journal.pone.0111954)
Supplement: Protocol S1 — (DOCX) [file pone.0111954.s002.docx]

# Enhancing activity, nutrition and mental health in overweight adolescents: Stage 2 Ethics Research Plan

**Aims/objectives of the study**

This study will use a wait-list controlled, staggered entry trial to assess the impact of a multidisciplinary intervention by:

1) comparing sedentary and moderate/vigorous activity before and after participation. We hypothesise that sedentary and moderate/vigorous activity will be improved after the program and this will be maintained at 3, 6 and 12 months post intervention.

2) comparing physical status before and after participation. We hypothesise that adiposity, cardiovascular fitness, muscle strength and power and coordination will be improved after the program and this will be maintained at 3, 6 and 12 months post intervention.

3) comparing food intake before and after participation. We hypothesise that intake of fruits and vegetables will be increased and intake of “extra” foods will be reduced after the program and this will be maintained at 3, 6 and 12 months post intervention.

4) comparing mental health status before and after participation. We hypothesise that mental health will be improved after the program and this will be maintained at 3, 6 and 12 months post intervention.

5) comparing perceived quality of life before and after participation. We hypothesise that quality of life will be improved after the program and this will be maintained at 3, 6 and 12 months post intervention.

We will also explore the influence of adolescent gender, baseline status, readiness to change, self efficacy, emotional eating and parental status and family function on changes in adolescent outcomes. Process evaluation will also be conducted to assess program fidelity, satisfaction and adverse outcomes.

**Background**

**Rationale for focusing on overweight adolescents**

The 2007 Children’s Nutrition and Physical Activity Survey found approximately 25% of boys and 30% of girls aged 9-13 years were classified as overweight or obese [1-2]. It has been suggested that changes to environmental and societal factors such as a decrease in physical activity, an increase in sedentary behaviour and the availability of high fat, high energy food have contributed to the high rates of overweight and obesity [3]. Obesity is seen as problematic in adolescents as it is related to short-term and long-term medical and psychosocial problems.  Short-term difficulties can include physical discomfort, hypertension, orthopaedic problems, sleep apnoea and increased risk of heart disease and Type II diabetes [4].  Psychosocial problems can include social isolation, decreased self-esteem and depression [5-6].

Adolescence has been identified as a critical period for the development of obesity [7]. Obese adolescents are more likely to become obese adults [8].  Longitudinal studies have indicated that individuals who remain overweight from adolescence to adulthood had lower economic, educational and social outcomes than normal weight peers even when initial socioeconomic factors were controlled for [9].

Despite the recognition of adolescence as a critical period in physical and psychosocial development, there is limited scientific interventions\ research with overweight adolescents. Glenny et al. [10] conducted an early meta-analysis of obesity treatment, and reviewed a total of 13 random-control trials with obese or overweight children or adolescents. They only considered studies with a minimum follow-up of 12 months, as maintenance of change was viewed as an important factor in the success of this type of intervention. They concluded there was a significant evidence gap with physical activity, lifestyle change and family involvement identified as promising foci for treatment. More recently, Oude et al. (2009) [11] conducted a Cochrane Review for treatment of obesity with both adolescents and children.  A total of 17 randomised controlled trials were reviewed with a focus on lifestyle interventions targeting physical activity, diet or behaviour therapy oriented treatments specifically for adolescents. Overall they concluded that *‘…family-based lifestyle interventions with a behavioural program aimed at changing diet and physical activity and thinking patterns provide significant and clinically meaningful decrease in overweight in ..adolescents..’*(p17). Whitlock et al. [12] review also concluded that recent evidence supports the efficacy of comprehensive programs of moderate to high intensity (>26hrs contact). A brief review of some of the supporting evidence is presented below (see also Stewart, Reilly & Hughes, 2008 [13]).

**Activity behaviours**

On an individual level, an imbalance between energy intake and energy expenditure can lead to obesity [14]. A change in energy balance is required to achieve weight loss outcomes, by either increasing energy expenditure through physical activity or reducing energy intake or ideally a combination of both. Achieving this outcome in adolescents is further complicated by the need to recognise that growth is still occurring. Kemper et al. [15] conducted a longitudinal study measuring body-mass index (BMI) and lifestyle factors from the age of 13 until 27 years. Their results demonstrated a strong relationship between high levels of physical activity and low body mass. Berkey, Rockett, Gillman and Colditz [16] conducted a large one-year prospective study and found that increased physical activity was associated with decreases in BMI in girls and overweight boys. Increases in sedentary behaviour were associated with increases in BMI.

The research above supports the utilisation of physical activity as a key component in adolescent obesity treatment. Interestingly the results from Anderson et al.,[17] suggested that targeting sedentary behaviour may significantly improve outcomes. Results from a study by Epstein et al. [18] support this proposition. In their randomised controlled trial with 8 – 12 year olds, participants were allocated to a treatment group which either targeted physical activity, sedentary behaviour or both. At twelve month follow-up the sedentary behaviour group had significantly greater weight reduction than the other two groups. These results have yet to be replicated with adolescent participants.

**Nutrition behaviours**

Obese adolescents appear to consume less well-balanced diets with more low nutrient, high energy foods consumed. Spear et al. [14] reviewed the evidence on various dietary prescriptions and concluded that there may be a relationship between increased overweight or obesity in adolescents and decreased fruit or fruit and vegetable consumption. Adolescents are low consumers of fruits and vegetables but consume 36 percent of their energy intake from unhealthy food or “junk food” [2, 19]. Available evidence supports interventions which provide education on nutritional and energy values of foods, and encouraged a healthier and more balanced diet, whilst discouraging dieting or restrictive eating [11]. Interventions that target dietary modification alone appeared to be less effective than those that combined dietary modification and physical activity [20]. Clearly an intervention strategy targeting both diet and physical activity and the motivators and barriers to weight maintenance would appear to be the best approach.

**Attitudes**

Spear et al. [14] argued that whilst most effective treatments for childhood obesity included both dietary and physical activity interventions, behavioural aspects also need to be considered. Behaviour modification treatment involves utilisation of behavioural change strategies aimed at changing thinking patterns and actions, which may be in relation to dietary intake, physical activity or other aims. Oude et al. [11] identified 12 studies that explored behavioural modification treatment with adolescents and performed a meta-analysis with four studies that fitted the criteria for this type of analysis. The meta-analysis revealed significant reduction in BMI at the end of treatment and maintenance of weight changes at 6 month follow-up compared with controls. The strategies that appeared most beneficial in behavioural management techniques were monitoring, goal-setting and contingency management [14].

Daley et al. [21] conducted a randomised control study with obese children aged 11-16 years. Participants were assigned to an exercise plus therapy condition, where motivation to change and behavioural modification were addressed whilst engaged in aerobic exercise, an exercise condition, involving light body conditioning only, or no treatment condition.  Participants in the behavioural treatment condition reported greater levels of self-esteem, although there were no changes to depression scores or BMI. Whilst behaviour therapy based treatment can produce positive outcomes, difficulties remain in relation to small weight reductions, maintenance and psychosocial problems. Brennan et al. (2008) argued that cognitive behaviour therapy (CBT) may be important in improving interventions for obese adolescents. Brennan et al. [22] conducted a randomised control study where CBT for weight loss was delivered on an individual basis to adolescents with their parents. They reported positive changes in weight loss and psychosocial measures. The current evidence supports the proposal that short-term CBT, in addition to behavioural strategies to address physical activity and eating behaviour, can provide positive outcomes in relation to weight changes and psychosocial stressors.

**Family**

Garn et al. [23] found that the offspring of two obese parents had an 80% probability of being obese compared with a 10% chance for offspring of two lean parents. Whilst it is acknowledged that there may be a genetic factor to the development of obesity, parental attitudes and behaviour also appear to have an impact [1]. Furthermore parents often remain responsible for food choices within the home for adolescents [1].

Stewart et al. [13] report that the expert consensus is that parents’ engagement in obesity treatment with children and adolescents is important. A recent example is where Andrews et al. [24] assessed 201 mothers and found maternal attitudes, social norms and perceived behavioural control predicted behavioural intentions and these in turn predicted parental tracking of child food behaviours.

**Summary**

Given the large proportion of Australian adolescents currently overweight it is clear current practices are insufficient. In order to avoid the expected trajectory to morbid obesity [25] Australian [26] and international [27] researchers have recommended urgent evaluation of interventions targeting overweight adolescents.

The recent evidence of the lack of efficacy of a GP delivered intervention for children [28] and promising early results from two multi-disciplinary community-based overweight adolescent programs [29-30] aligns with earlier expert panel recommendations for “comprehensive multi-disciplinary intervention…[to] encourage healthy behaviours while using techniques to motivate patients and families..” [14] (p.254). The most recent systematic reviews [11-12] have confirmed this approach. Oude et al [11] identified critical issues for future research including: interventions for different levels of severity (we will target overweight and mildly obese), strategies for long-term maintenance (we will work with communities and families to identify sustainability factors), family characteristics which promote success (our process assessment with collect this), adolescent psychosocial factors which influence change (our broad evaluation framework will capture this), resource-effective methods for delivering interventions in different settings (we will evaluate this in metropolitan and regional community settings), and potential harm and benefits of intervention (we will monitor adverse effects as well as health outcomes).

Given the current evidence and strong recommendations for urgent action on this ‘global epidemic’ [11] this project will build on published evidence, recent pilot work and team experience to refine and implement a multi-disciplinary family-centred community-based intervention intended to influence the physical activity, nutrition and psychosocial behaviours of overweight adolescents

**Methods**

**Intervention:** The intervention will be based on a successful pilot program conducted by the research team that in turn was based on current evidence for the management of adolescent obesity [11, 30-31] and the successful “Fitmatters” program conducted in a tertiary clinical setting. Key strategies of the intervention were: focus on healthy behaviours rather than weight management, multi-disciplinary facilitators and family involvement. The intervention also focussed on the psychosocial aspects of being overweight and was based on to the families’ readiness to change using the PRECEDE-PROCEED Framework [32-35]

The program consists of an 8 week multi-disciplinary family-centred intervention focussed on improving activity, food and attitude habits. Groups of up to 12-15 adolescents and their parents attended 2 hour sessions twice weekly. On each occasion adolescents participated in a 45 minute exercise class involving aerobic, strength and coordination stations. Adolescents also participated in education sessions covering moderate physical activity, sedentary behaviours and diet. Tailored feedback on fitness, screen time and diet were provided to each adolescent. Other sessions provided support and group counselling on setting goals, overcoming set-backs and dealing with cognitive and emotional issues. Adolescents were set ‘homework’ relevant to the stage in the program – for example keeping a diary of TV programs watched. Parents participated in education sessions covering the same issues as the adolescents, sometimes with their child and sometimes separately. Parents also gained informal support from other parents and staff in ‘walk and talk’ sessions, which itself modelled a way of building more activity into their day. Parents were also given practical training in buying and preparing healthy food during a supermarket visit and cooking classes. All sessions were run during school term but out of school hours. A brief program outline is shown in Table 1 (each week has two rows – one for each program day). We intend to include regular contact (SMS, phone, mail) to assist motivation and maintenance of effects after the 8 weeks.

# Study design: A wait-list controlled, staggered-entry cohort design will be used. Participants will be recruited and given an initial assessment then reassessed after 3 months waiting (just before starting the program). The dual pre-participation assessments will provide a within-subjects control period. Follow-up assessments will be conducted immediately after completing the program (3 months after second pre-program assessment) and at 3, 6 and 12 months post program.

Cohorts of 12-15 overweight adolescents and their parents will be progressively recruited over the study period. Cohorts are expected to start in Perth metropolitan October 2011, February 2012 and April 2012. The staggered start will control for external seasonal and public event confounders to intervention effects.

Table 1. Project community engagement (*), implementation cohorts (shaded) and follow up assessments (x)

| **Cohort** | **2011** | | | | **2012** | | | | **2013** | | | |
| --- | --- | --- | --- | --- | --- | --- | --- | --- | --- | --- | --- | --- |
|  | 1/4 | 2/4 | 3/4 | 4/4 | 1/4 | 2/4 | 3/4 | 4/4 | 1/4 | 2/4 | 3/4 | 4/4 |
| South metro | * | * | recruit | waitlist | S1 | S2 | S3x | x | x | x | x |  |
| East metro | * | * | recruit | waitlist | N1 | N2 | N3x | x | x | x | x |  |
| Regional | * |  | * | * | recruit | waitlist | R1 | R2 | x | x | x | x |

# Participant recruitment procedures: Adolescents and their families will be recruited via the health system, the education system and from the general community. Paediatric specialists and allied health professionals will be asked to identify potentially suitable adolescents. Print and radio mass media will be used to inform the general community of the study. In addition to these sources, participants will be recruited from workplaces that employ a large number of adolescents, within the Perth metropolitan area using the snowball technique [36]. Volunteers will be screened to ensure they meet the inclusion and exclusion criteria. Inclusion criteria will be: males and females aged 12-16 years; body mass index higher than the 85^th^ percentile (includes children who are typically classified as overweight or obese; [37]). Children will be excluded if they: are obese due to identified genetic factors; have morbid obesity (BMI z score higher than 3.5); have a metabolic/endocrine disease; are unable to attend twice weekly sessions, are undergoing treatment for psychiatric disorders or are assessed as unsafe to participate. Volunteers will be approved for participation by their medical practitioner.

**Sample size:** A sample size of 96 is required at post test in the intervention group to detect a 20% difference in the outcome variables at 80% power and 5% level of significance. A small effect size (0.2) is assumed for studies on behavioural effects due to the influence of extraneous variables and the subtleties of human performance. Allowing for an attrition rate of 30%, 124 adolescents and their families will be recruited into the intervention.

**Evaluation**

There is substantial agreement that interventions for overweight young people need to be evaluated in terms of process and outcome and not just immediate impact (Livingstone et al 2007) to understand not only how much the intervention works but why and for whom.

**Process evaluation:** A broad process evaluation will be conducted in line with the recent framework for mandatory evaluation to ensure well informed public policy decisions [38] and recommendations by the recent Cochrane review for obesity interventions [11]. Questionnaire surveys of participants, focus groups of participants and program facilitators are anticipated [39].

Program fidelity will be assessed through observations of sessions, review of program notes and focus groups with program staff [40]. Program dose will be assessed by attendance records. Barriers to participation will be explored through participant/parent surveys including reasons for non attendance and gaining reasons for drop out from those who do not complete the program [41]. Participant satisfaction will be assessed with a validated tool [42] along with focus groups. Monitoring will also be conducted for potential adverse effects, specifically: changes in linear height, disordered eating, and psychological well being [11].

**Impact evaluation:** Immediate impact of the intervention will be assessed by comparing the changes over the 3 months between pre program assessment and immediately post program assessment with any changes over the 3 months ‘wait-list’ period between initial assessment and pre program assessment.

The primary impact measures will be changes in: 1) leisure time spent in light and moderate/vigorous physical activity; 2) leisure time spent in sedentary activity; 3) number of weekly serves of fruit and vegetables and 4) number of weekly serves of extras.

Secondary impact measures will include: readiness to change state, exercise self efficacy and food self efficacy, meeting of sedentary screen time, vigorous physical activity and key diet target behaviours.

**Outcome evaluation:** The longer term changes in behaviour (same variables as short term impact), as well as physical and mental well being and family functioning, will be assessed by comparing changes between assessments at baseline and at 3, 6 and 12 months post intervention.

Physical status: Adiposity, cardiovascular fitness, muscle strength and power and motor competence will be assessed. Mental status: Attitudes to PA, moods and feelings, self perception and quality of life will be assessed. Family functioning will also be assessed.

**Expected program outcomes:** The primary program outcomes are expected to be improved adolescent behaviours including: an increase in physical activity (10% change)**;** reduction in sedentary behaviours (10% change)**;** an increase in fruit and vegetable serves (10% change)**;** a reduction in “junk food” serves (high fat, high sugar foods) (10% change)**.** We also anticipate improved physical health status; improved mental health including anxiety, depression, self worth, perceived physical appearance, physical ability and social support**;** improved quality of life and improved family functioning. We also anticipate improved parental mental health.

**Instruments: Outcome measures for adolescent**

## *Sedentary activity*

Weekly frequency and school day/weekend day duration of participation in common sedentary behaviours (TV viewing, playing electronic games, using a computer) and moderate/vigorous physical activity (sports, dance, active play) will be assessed using questions drawn from the Western Australian Child and Adolescent Physical Activity and Nutrition Survey and the Health Kids Queensland Survey [43-44].

## *Moderate/Vigorous Physical activity*

Time spent in moderate to vigorous intensity physical activity (PA) will be assessed over 7 days using a Actical accelerometer and pedometer worn on the hip. The MiniMitter Actical is the most widely used and validated accelerometer in studies of children and adolescents [45-46]. Seven days of accelerometer and pedometer measurement are recommended for the purposes of acceptable measurement of moderate to vigorous PA [46]. Total weekly PA as well as weekend PA and after school weekday PA will be assessed.

## *Food intake*

It is acknowledged that all methods of dietary data collection are fraught with limitations [47] and adolescents are known to be a particularly hard group from which to collect detailed and accurate dietary records [48-49]. Overweight adolescents tend to underreport their intake because of factors including lack of motivation, body image concerns, rebellion against authority and unstructured eating habits that make recording difficult [48, 50-51]. It is recommended that intervention studies for overweight participants use at least two types of dietary assessment tools [52] and for this study, adolescent nutrition will be assessed through collection of data from three day food records using weighed or household measures and an eating behaviour checklist that collects data on key eating behaviours. In national surveys 24 hour recalls are commonly used, however, in adolescents this measure tends not to reflect the wide variation in teen diets over several days [49]. Three day food records are the most appropriate tool in this instance to measure intake and allow for analysis of detailed nutrition information from a small sample group. The burden of recording for adolescents has been considered and food records have therefore been kept to three days to maximise participation. Food diary analysis will provide information about the number of serves of fruits, vegetables and extra foods, as described by the Australian Guide to Healthy Eating [53]. The total energy intake and intake of macronutrients and micronutrients will be determined using the AUSNUT database and Foodworks Professional edition version 3.02 software. Participants will also complete the eating behaviour checklist to provide information about their eating behaviours and perceived intake. This checklist will be used to give feedback to adolescents and based on questionnaires used in similar cohorts [54]. Questions include whether participants eat breakfast, how often they eat fast food and how often they eat meals with their family. All analysis will be completed by a qualified dietitian.

*Adiposity*

Weight, height and waist circumference measurements will be made using calibrated scales, stadiometer and inelastic tape measure and standardized protocols. Body mass index will be calculated and age and sex adjusted BMI z-scores determined to enable comparison with state-wide surveys (WA, QLD).

## *Fitness*

Cardiovascular fitness will be assessed using the modified incremental shuttle walk test [55] (University Hospitals of Leicester NHS trust, Leicester, UK). This test is based on the widely used incremental shuttle run test designed for healthy individuals [56], with the reduction of shuttle distance from 20m to 10m to provide a lower demand start to the test for those with health problems. Participants walk/run between markers in time with beeps. The beep interval decreases every minute, requiring increases in walking velocity. The test is terminated when the participant can no longer reach the end of the 10m in time with the beep. In obese adolescents test-rest reliability is very high (ICC=0.92) and validity has been demonstrated with strong correlations with other aerobic indices including maximal oxygen uptake in bicycle ergonometer tests (r=0.79) [57].

Strength of the quadriceps, biceps and deltoid muscles will be assessed using standard ‘break’ manual muscle testing protocols [58]. ‘Break’ tests require the participant to hold a position while the tester applies progressive resistance until the position is broken. Peak force will be recorded using a force transducer. All muscle testing will be performed on the non-dominant limb and each test will be performed 3 times, with an average score used for analysis. The testing positions for the myometer were modified from Backman et al. [59]. For quadriceps (knee extensors), participants will sit upright in a chair with thighs supported and hands rested lightly on the thighs, palms up. The participant will be directed to extend their non-dominant knee fully and the Myometer will be applied to the anterior lower limb just above the malleoli. The participant will try and maintain that position as the researcher applies resistance in the opposite direction. The dominant lower limb must remain still at all times [59]. For biceps (elbow flexors), participants will be positioned with their dominant arm relaxed by their side, with the non dominant hand in 90° of elbow flexion and supinated, The researcher will stabilise the non-dominant arm posteriorly superior to the elbow, ensuring the elbow remains free to move. The Myometer will be applied on the forearm just proximal to the radial styloid process. The participant will be instructed to keep their elbow flexed at 90° as resistance is provided by the researcher in the opposite direction.[59] For deltoids (shoulder abductors), participants will be seated with the non-dominant shoulder abducted to 90°, elbow extended, forearm in mid pronation with palm facing down. The dominant arm remains rested on the participant’s lap. The researcher will stabilise the non- dominant shoulder, ensuring the shoulder remains free to move. The Myometer will be applied to the upper limb approx 2cm proximal to the lateral humeral epicondyle. The participant will be instructed to maintain the testing position as resistance is provided by the researcher in the opposite direction [59]. Hand held force transducer tests of muscle strength have been shown to have good criterion validity in comparison to laboratory dynamometers, with good intra-tester and inter-tester reliability [60].

Muscle power will be assessed using a vertical jump protocol [61]. The participant will stand side on to the wall and reach as high as possible with their hand closest to the wall, feet flat on the ground; the height of the finger tips is measured. The participant then jumps vertically as high as possible using a knee bend and arm swing motion to assist in projecting the body. The participant attempts to touch the wall at the highest point of the jump. The distance between the standing reach height and the jump reach height is recorded. Three measures are taken and the highest recorded. Reliability has been reported to be high (ICC = 0.96) [62]. Vertical jump has been used to determine power outputs in obese adolescents [63].

## *Mental health*

The primary outcome measure will be depressive symptoms assessed using the Moods and Feelings questionnaire (MFQ) [64]. The MFQ is a 33 item self-report measure which looks at depressive symptoms in children and adolescents. It has good test-retest reliability [65] and has been validated with a clinical population [66].

*Motor coordination*

Motor competence will be assessed using Movement Assessment Battery for Children-2 (MABC-2 ) [67]. The MABC-2 comprises 8 tasks, three measuring manual dexterity, 3 measuring aiming and catching and 2 measuring balance. Age norms are used to determine a standard score based on a distribution with a mean of 10 and a standard deviation of 3. Separate standard scores can be determined for each of the sub-tests. Minimum value of the test-retest reliability of the original MABC is 0.75 and the inter-tester reliability is 0.70. The MABC has been found to correlate well with other movement tests [68-69].

## *Activity and food guideline behaviour, readiness to change and self efficacy*

Meeting of sedentary screen time, vigorous physical activity and key diet behaviours and readiness to change if not currently meeting recommended behaviours will be assessed using questions used in an adult obesity study [70] based on the transtheoretical model of health behaviour change [71]. This approach has been used successfully for childhood obesity [72] and the item have demonstrated good concurrent validity [70].

Self efficacy in relation to confidence in being able to exercise for 60 minutes every day and eat a healthy diet on a regular basis will be assessed using Bandura’s exercise self efficacy [73] and weight efficacy lifestyle [74] scales. The scales consist of 18 and 20 items respectively and cover different situational domains such as negative emotions, social pressure, physical discomfort and availability/external constraints. Participants rate their confidence in being able to maintain desired behaviour on a ten point scale from 0 (not certain) to 9 (very certain). The scales were slightly modified to reflect the target behaviours of the study as recommended [75]. The exercise self efficacy scale has demonstrated good psychometric properties, sensitivity to change over a 6 week intervention and correlation with objective functional status measures [73]. The weight efficacy lifestyle scale has demonstrated good internal consistency, sensitivity to change with a nine week intervention, and significant correlations with a related eating self efficacy scale [74].

*Attitudes to PA, Eating and Self*

Attitudes to PA will be assessed using the revised Physical Activity Enjoyment Scale (PAES) [76]. Internal consistency, test-retest reliability and construct validity has been demonstrated [77-79].

Attitudes to eating will be assessed with the Dutch Eating Behaviours Questionnaire (DEBQ) [80]. The DEBQ is a 33 item self-report measure of eating styles developed to increase understanding of eating patterns in obese individuals. The measure has three scales, restrained, external and emotional eating. This measure has been shown to have good internal consistency, discriminative and concurrent validity and factor stability [80-81]. This measure has also been demonstrated as appropriate for use in children over 12 years of age [82].

Harter’s Self-Perception Profile for Children (SPPC) [83] is a 36 item self-report measure of self-concept. The SPPC comprises five subscales; Scholastic Competence, Social Acceptance, Athletic Competence, Physical Appearance, and Behavioural Conduct; and one global self-worth scale. The Athletic Competence, Physical Appearance and Global Self-worth subscales will be used in the current study. It has been validated in samples of children from a wide range of cultural backgrounds, including in Australian children and has high levels of internal consistency. Harter [83] reported factor-analytic data that confirmed the use of these subscales and good internal reliabilities which have been replicated in other studies [84].

## *Quality of life*

Quality of life will be assessed with the Paediatric Quality of Life – Teen Report (PedsQL) [85]. The PedsQL is a 23-item self-report quality of life measure for 13-18 year olds. This measure has been demonstrated to have good validity and reliability [86].

## *Additional information*

Socioeconomic status will be characterised by the Index of Relative Socioeconomic Advantage Disadvantage (IRSAD) developed by the Australian Bureau of Statistics (ABS) using 2001 national census data. The IRSAD is a general socio-economic index based on 21 measures of both disadvantage and advantage including annual household income, persons over 15 educational attainment, employment status, unskilled vs. skilled occupations, number of bedrooms in dwelling, percentage of one parent families, value of weekly rent, private dwelling with no car, ownership of residing dwelling, value of weekly mortgage repayments, private dwellings with internet and number renting from government or not for profit agency. Scores with low index values indicate areas of disadvantage and high values indicate areas of advantage. Ethnicity will also be recorded.

**Instruments: Outcome measures for parent**

Parents will complete the several of the same assessments (activity questionnaire, meeting guidelines questionnaire, physical activity enjoyment questionnaire) along with adult versions of questionnaires covering the moods and feelings area (DASS-2121). The DASS-21 is a short-form of the Depression, Anxiety and Stress Scale (DASS) [87]. The DASS has been shown to reliably distinguish between symptoms of depression, anxiety and stress in clinical [88] and non-clinical samples [87]. The DASS-21 has been demonstrated to have acceptable reliability and validity [89].

*Family Outcome Measure*

Parents will also complete the general functioning scale of the McMaster Family Assessment Device (FAD) [90]. The FAD is a 53 item self-report which assesses family functioning on five subscales; problem-solving, affective responsiveness, affective involvement, behaviour control and general functioning. The FAD is suitable to be administered to all members of a family over 12 years, and has adequate internal consistency, test-retest reliability and concurrent validity [90-91].

Families will be interviewed on site and invited to discuss their family lifestyles, including discourses of gender, ethnicity, socioeconomic status and education; attitudes to food, eating and exercise in order to identify challenges and barriers to change. These activities will be conducted in collaboration with the research team to identify and explore means of achieving change for the adolescents and families. Families will be visited at home to observe lifestyle and potential resources in achieving change. These qualitative assessments will augment the results of the quantitative measures, and provide the opportunity to discuss discrepancies.

## Analysis plan

Process evaluation will use descriptive statistics and qualitative analysis.

Impact and outcome evaluation will use a multi-level mixed modelling approach for the hierarchical data collected over the one-year observational period (repeated measurements of individuals nested within families.) Estimates of change in outcomes over time periods will be adjusted for potential confounders of age, gender, maturation level, IRSAD and parental education levels. All change estimates will be presented as adjusted mean change with corresponding 95% confidence intervals. Non-normally distributed data will be appropriately transformed as necessary, and statistical models checked for unduly influential outliers. All data analysis will be performed utilising current statistical software (Stata/IC 10.1 for Windows, Statacorp).

**Significance/justification of the study**

The activity, nutrition and psychosocial well being of young people is important for the future health of Australians, particularly if the emergence of significant levels of overweight and obesity in youth are to be addressed. Given that overweight adolescents are much more likely to become overweight or obese adults, with a concomitantly higher likelihood of morbidity, the need for better information to inform best practice and improve the health outcomes of this important group is clear. It is expected that the project will provide a stand-alone program that can be rolled out across Australia at a community level by appropriately trained community-based personnel.

References

1. National Health and Medical Research Council, *Clinical practice guidelines for the management of overweight and obesity in children and adolescents*, NHMRC, Editor. 2003: Canberra.

2. Department of Health and Ageing, Australian Food and Grocery Council, and Department of Agriculture Fisheries and Forestry, *2007 Australian National Children's Nutrition and Physical Activity Study*. 2007.

3. Baur, L.A., *Child and adolescent obesity in the 21st century: an Australian perspective.* Asia Pacific Jorunal of Clinical Nutrition, 2002. **11**(Suppl 3): p. S524-S528.

4. Steinbeck, K., *Childhood obesity. Treatment options.* Best Pract Res Clin Endocrinol Metab, 2005. **19**(3): p. 455-69.

5. Merten, M., K. Wickrama, and A. Williams, *Adolescent Obesity and Young Adult Psychosocial Outcomes: Gender and Racial Differences.* Journal of Youth and Adolescence, 2008. **37**(9): p. 1111.

6. Strauss, R., *Childhood obesity and self-esteem.* Pediatrics, 2000. **105**(1): p. e15.

7. Dietz, W., *Critical periods in childhood for the development of obesity.* The American Journal of Clinical Nutrition, 1994. **59**(5): p. 955-959.

8. Guo, S.S., et al., *Predicting overweight and obesity in adulthood from body mass index values in childhood and adolescence.* Am J Clin Nutr, 2002. **76**(3): p. 653-8.

9. Gortmaker, S.L., et al., *Social and economic consequences of overweight in adolescence and young adulthood.* N Engl J Med, 1993. **329**(14): p. 1008-12.

10. Glenny, A.M., et al., *The treatment and prevention of obesity: a systematic review of the literature.* Int J Obes Relat Metab Disord, 1997. **21**(9): p. 715-37.

11. Oude Luttikhuis, H., et al., *Interventions for treating obesity in children.* Cochrane Database Syst Rev, 2009(1): p. CD001872.

12. Whitlock, E., et al., *Effectiveness of weight management interventions in children: A targeted systematic review for the USPSTF.* Pediatrics, 2010. **125**: p. e396-e418.

13. Stewart, L., J.J. Reilly, and A.R. Hughes, *Evidence-based behavioral treatment of obesity in children and adolescents.* Child Adolesc Psychiatr Clin N Am, 2009. **18**(1): p. 189-98.

14. Spear, B.A., et al., *Recommendations for treatment of child and adolescent overweight and obesity.* Pediatrics, 2007. **120 Suppl 4**: p. S254-88.

15. Kemper, H.C., et al., *Lifestyle and obesity in adolescence and young adulthood: results from the Amsterdam Growth And Health Longitudinal Study (AGAHLS).* Int J Obes Relat Metab Disord, 1999. **23 Suppl 3**: p. S34-40.

16. Berkey, C.S., et al., *One-Year Changes in Activity and in Inactivity Among 10- to 15-Year-Old Boys and Girls: Relationship to Change in Body Mass Index.* Pediatrics, 2003. **111**(4): p. 836-843.

17. Andersen, R.E., et al., *Relationship of physical activity and television watching with body weight and level of fatness among children: results from the Third National Health and Nutrition Examination Survey.* JAMA, 1998. **279**(12): p. 938-42.

18. Epstein, L.H., et al., *Effects of decreasing sedentary behavior and increasing activity on weight change in obese children.* Health Psychology, 1995. **14**(2): p. 109-115.

19. Cook, T., I. Rutishauser, and M. Seelig, *Comparable data on food and nutrient intake and physical measurements from the 1983, 1985 and 1995 National Nutrition Surveys*, Australian Food and Nutrition Monitoring Unit, Editor. 2001, Commonwealth Department of Health and Ageing: Canberra.

20. Epstein, L.H., et al., *Effect of diet and controlled exercise on weight loss in obese children.* J Pediatr, 1985. **107**(3): p. 358-61.

21. Daley, A.J., et al., *Exercise therapy as a treatment for psychopathologic conditions in obese and morbidly obese adolescents: A randomized, controlled trial.* Pediatrics, 2006. **118**(5): p. 2126-2134.

22. Brennan, L., et al., *Motivational interviewing and cognitive behaviour therapy in the treatment of adolescent overweight and obesity: Study design and methodology.* Contemporary Clinical Trials, 2008. **29**(3): p. 359-375.

23. Garn, S.M. and D.C. Clark, *Trends in fatness and the origins of obesity Ad Hoc Committee to Review the Ten-State Nutrition Survey.* Pediatrics, 1976. **57**(4): p. 443-56.

24. Andrews, K., K. Silk, and I. Eneli, *Parents as health promoters: A theory of planned behavior perspective on the prevention of childhood obesity.* Journal of Health Communication, 2010. **15**(1): p. 95-107.

25. Swinburn, B., et al., *Obesity Prevention Programs Demand High-Quality Eveluations.* Aust NZ J Public Health, 2007. **31**: p. 305-307.

26. Denney-Wilson, E. and L. Baur, *Adolescent obesity: Making a difference to the epidemic.* International Journal of Adolescent Medicine and Health, 2007. **19**(3): p. 235-243.

27. Eneli, I.U., A. Cunningham, and S.J. Woolford, *The pediatric multidisciplinary obesity program: An update.* Progress in Pediatric cardiology, 2008. **25**: p. 129-136.

28. Wake, M., et al., *Outcomes and costs of primary care surveillance and intervention for overweight or obese children: the LEAP 2 randomised controlled trial.* BMJ, 2009. **339**: p. b3308.

29. Evans, R.K., et al., *Evaluation of a 6-month multi-disciplinary healthy weight management program targeting urban, overweight adolescents: Effects on physical fitness, physical activity, and blood lipid profiles.* International Journal of Pediatric Obesity, 2009. **4**(3): p. 130-133.

30. Shrewsbury, V., et al., *A randomised controlled trial of a community-based healthy lifestyle program for overweight and obese adolescents: the Loozit® study protocol.* BMC Public Health, 2009. **9**(1): p. 119.

31. Jones, R., et al., *The HIKCUPS trial: a multi-site randomized controlled trial of a combined physical activity skill-development and dietary modification program in overweight and obese children.* BMC Public Health, 2007. **7**(1): p. 15.

32. Glanz, K., B. Rimer, and F. Lewis, *Health behaviour and health education: theory, research and practice*. 3rd ed. 2002, San Fransisco: Jossey-Bass.

33. Green, L. and M. Kreuter, *Health Promotion Planning: An Educational and Ecological Approach* 3rd ed. 1999, Mountain View, CA: Mayfield Publishing Co.

34. Green, L. and M. Kreuter, *Health Program Planning: An Educational and Ecological Approach*. 4th ed. 2005, New York: McGraw Hill.

35. Howat, P., et al., *Population health, community and health promotion*, ed. S. Jirojwong and P. Liamputtong. 2008: Oxford University Press.

36. Hawe, P., D. Degeling, and J. Hall, *Evaluating Health Promotion*. 1994, Sydney: MacLennan and Petty.

37. Cole, T.J., et al., *Establishing a standard definition for child overweight and obesity worldwide: international survey.* BMJ, 2000. **320**(7244): p. 1240-3.

38. Oxman, A.D., et al., *A framework for mandatory impact evaluation to ensure well informed public policy decisions.* Lancet, 2010. **375**(9712): p. 427-31.

39. Oakley, A., et al., *Process evaluation in randomised controlled trials of complex interventions.* British Medical Journal, 2006. **332**(7538): p. 413-416.

40. Schneider, M., et al., *Rationale, design and methods for process evaluation in the HEALTHY study.* International journal of obesity, 2009. **33**(Suppl 4): p. S60-S67.

41. Robertson, W., et al., *Pilot of" Families for Health": community-based family intervention for obesity.* Archives of Disease in Childhood, 2008. **93**(11): p. 921.

42. Tarnowski, K.J., et al., *Acceptability of Treatments for Child Behavioral Disturbance - Race, Socioeconomic-Status, and Multicomponent Treatment Effects.* Child & Family Behavior Therapy, 1992. **14**(1): p. 25-37.

43. Hands, B., et al., *Physical Activity and Nutrition Levels in Western Australian Children and Adolescents*. 2004, Western Australian Government: Perth.

44. Abbott, R., et al., *Healthy Kids Queensland Survey 2006 - Full Report*. 2008, Queensland Health: Brisbane.

45. Puyau, M.R., et al., *Validation and calibration of physical activity monitors in children.* Obes Res, 2002. **10**(3): p. 150-7.

46. Trost, S.G., K.L. McIver, and R.R. Pate, *Conducting accelerometer-based activity assessments in field-based research.* Med Sci Sports Exerc, 2005. **37**(11 Suppl): p. S531-43.

47. Collins, C.E., J. Watson, and T. Burrows, *Measuring dietary intake in children and adolescents in the context of overweight and obesity.* Int J Obes, 2010. **34**(7): p. 1103-1115.

48. Livingstone, M.B.E. and A.E. Black, *Markers of the validity of reported energy intake.* Journal of Nutrition, 2003. **133**(3): p. 895s-920s.

49. Livingstone, M.B.E. and P.J. Robson, *Measurement of dietary intake in children.* Proceedings of the Nutrition Society, 2000. **59**(2): p. 279-293.

50. Livingstone, M., T. McCaffrey, and K. Rennie, *Childhood obesity prevention studies: lessons learned and to be learned.* Public Health Nutrition, 2007. **9**(8A): p. 1121-1129.

51. Boushey, C.J., et al., *Use of technology in children's dietary assessment.* European Journal of Clinical Nutrition, 2009. **63**: p. S50-S57.

52. Kristal, A.R., S.A. Beresford, and D. Lazovich, *Assessing change in diet-intervention research.* Am J Clin Nutr, 1994. **59**(1 Suppl): p. 185S-189S.

53. Smith, A., E. Kellett, and Y. Schmerlaib, *The Australian guide to healthy eating: background information for nutrition educators*. 1998, Commonwealth Department of Health and Family Services: Canberra.

54. Martin, K., et al., *Move and Munch Final Report. Trends in physical activity, nutrition and body size in Western Australian children and adolescents: the Child and Adolescent Physical Activity and Nutrition Survey (CAPANS)*. 2008.

55. Bradley, J., et al., *Validity of a modified shuttle test in adult cystic fibrosis.* Thorax, 1999. **54**(5): p. 437-439.

56. Leger, L.A. and J. Lambert, *A maximal multistage 20-m shuttle run test to predict VO2 max.* Eur J Appl Physiol Occup Physiol, 1982. **49**(1): p. 1-12.

57. Klijn, P.H.C. and O.H. van der Baan-Slootweg, *Aerobic exercise in adolescents with obesity: preliminary evaluation of a modular training program and the modified shuttle test.* BMC Pediatrics, 2007. **7**: p. 19.

58. Sloan, C., *Review of the reliability and validity of myometry with children.* Physical & Occupational Therapy in Pediatrics, 2002. **22**(2): p. 79-93.

59. Backman, E., et al., *Isometric muscle force and anthropometric values in normal children aged between 3.5 and 15 years.* Scandinavian journal of rehabilitation medicine, 1989. **21**(2): p. 105-114.

60. Li, R.C., et al., *The Development, Validity, and Reliability of a Manual Muscle Testing Device With Integrated Limb Position Sensors.* Archives of Physical Medicine and Rehabilitation, 2006. **87**(3): p. 411-417.

61. Klavora, P., *Vertical-jump tests: A critical review.* Strength and Conditioning Journal, 2000. **22**(5): p. 70-74.

62. Markovic, G., et al., *Reliability and factorial validity of squat and counter movement jump tests.* Journal of Strength and Conditioning Research, 2004. **18**(3): p. 551-555.

63. Achard de Leluardiere, F., et al., *Validation and influence of anthropometric and kinematic models of obese teenagers in vertical jump performance and mechanical internal energy expenditure.* Gait Posture, 2006. **23**(2): p. 149-58.

64. Costello, E.J. and A. Angold, *Scales to assess child and adolescent depression: checklists, screens, and nets.* J Am Acad Child Adolesc Psychiatry, 1988. **27**(6): p. 726-37.

65. Wood, A., et al., *Properties of the mood and feelings questionnaire in adolescent psychiatric outpatients: a research note.* J Child Psychol Psychiatry, 1995. **36**(2): p. 327-34.

66. Daviss, W.B., et al., *Criterion validity of the Mood and Feelings Questionnaire for depressive episodes in clinic and non-clinic subjects.* Journal of Child Psychology and Psychiatry, 2006. **47**(9): p. 927-934.

67. Henderson, S.E., D.A. Sugden, and A.L. Barnett, *Movement Assessment Battery for Children-2*. 2007, London, UK: Harcourt Assessment.

68. Laszlo, J.I. and P.J. Bairstow, *Control of skilled movement: processes underlying acquisition and performance. Perceptual-motor behaviour: Developmental assessment and therapy*. 1985, London: Holt, Rinehart & Winston.

69. Smits-Engelsman, B.C.M., S.E. Henderson, and C.G.J. Michels, *The assessment of children with Developmental Coordination Disorders in the Netherlands: The relationship between the Movement Assessment Battery for Children and the Korperkoordinations Test fur Kinder.* Human Movement Science, 1998. **17**(4-5): p. 699-709.

70. Robinson, A.H., et al., *Validating stage of change measures for physical activity and dietary behaviors for overweight women.* International journal of obesity, 2008. **32**(7): p. 1137-1144.

71. Prochaska, J.O., C.C. DiClemente, and J.C. Norcross, *In search of how people change: Applications to addictive behaviors.* Journal of Addictions Nursing, 1993. **5**(1): p. 2-16.

72. Beckman, H., S. Hawley, and T. Bishop, *Application of theory-based health behavior change techniques to the prevention of obesity in children.* Journal of Pediatric Nursing, 2006. **21**(4): p. 266-275.

73. Everett, B., Y. Salamonson, and P. Davidson, *Bandura's exercise self-efficacy scale: Validation in an Australian cardiac rehabilitation setting.* International Journal of Nursing Studies, 2009. **46**(6): p. 824-829.

74. Clark, M., et al., *Self-efficacy in weight management.* Journal of Consulting and Clinical Psychology, 1991. **59**(5): p. 739-744.

75. Bandura, A., *Guide for constructing self-efficacy scales*, in *Self-efficacy beliefs of adolescents*, F. Pajares and T. Urdan, Editors. 2006, Information Age Publishing: Greenwich, CT. p. 307-337.

76. Moore, J., et al., *Measuring enjoyment of physical activity in children: validation of the Physical Activity Enjoyment Scale.* Journal of Applied Sport Psychology, 2009. **21**(Supplement 1): p. S116-S129.

77. Kendzierski, D. and K.J. Decarlo, *Physical-Activity Enjoyment Scale - 2 Validation Studies.* Journal of Sport & Exercise Psychology, 1991. **13**(1): p. 50-64.

78. Motl, R.W., et al., *Measuring enjoyment of physical activity in adolescent girls.* American Journal of Preventive Medicine, 2001. **21**(2): p. 110-117.

79. Dishman, R.K., et al., *Enjoyment mediates effects of a school-based physical-activity intervention.* Medicine & Science in Sports & Exercise, 2005. **37**(3): p. 478-487.

80. van Strien, T., et al., *The Dutch Eating Behaviour Questionnaire (DEBQ) for assessment of restrained, emotional, and external eating.* International Journal of Eating Disorders, 1986. **5**: p. 295-315.

81. Stice, E., K. Presnell, and D. Spangler, *Risk factors for binge eating onset in adolescent girls: a 2-year prospective investigation.* Health Psychol, 2002. **21**(2): p. 131-8.

82. Braet, C., et al., *Differences in eating style between overweight and normal-weight youngsters.* J Health Psychol, 2008. **13**(6): p. 733-43.

83. Harter, S., *The Self-Perception Profile for Children: Revision of the perceived competence scale for children*. 1985, University of Denver: Denver, US.

84. Granleese, J. and S. Joseph, *Reliability of the Harter Self-Perception Profile for Children and predictors of global self-worth.* J Genet Psychol, 1994. **155**(4): p. 487-92.

85. Varni, J.W., M. Seid, and P.S. Kurtin, *PedsQL 4.0: reliability and validity of the Pediatric Quality of Life Inventory version 4.0 generic core scales in healthy and patient populations.* Med Care, 2001. **39**(8): p. 800-12.

86. Varni, J.W., et al., *Longitudinal factorial invariance of the PedsQL 4.0 Generic Core Scales child self-report Version: one year prospective evidence from the California State Children's Health Insurance Program (SCHIP).* Qual Life Res, 2008. **17**(9): p. 1153-62.

87. Lovibond, S.H. and P.E. Lovibond, *Manual for the Depression Anxiety Stress Scales*. 2nd ed. 1995, Sydney, Australia: Psychology Foundation of Australia.

88. Brown, T.A., et al., *Psychometric properties of the Depression Anxiety Stress Scales (DASS) in clinical samples.* Behav Res Ther, 1997. **35**(1): p. 79-89.

89. Antony, M.M., et al., *Psychometric properties of the 42-item and 21-item versions of the Depression Anxiety Stress Scales in clinical groups and a community sample.* Psychological Assessment, 1998. **10**(2): p. 176-181.

90. Epstein, N.B., L.M. Baldwin, and D.S. Bishop, *The Mcmaster Family Assessment Device.* Journal of Marital and Family Therapy, 1983. **9**(2): p. 171-180.

91. Miller, I.W., et al., *The Mcmaster Family Assessment Device - Reliability and Validity.* Journal of Marital and Family Therapy, 1985. **11**(4): p. 345-356.
